# Gene knockout of *Zmym3* in mice arrests spermatogenesis at meiotic metaphase with defects in spindle assembly checkpoint

**DOI:** 10.1038/cddis.2017.228

**Published:** 2017-06-29

**Authors:** Xiangjing Hu, Bin Shen, Shangying Liao, Yan Ning, Longfei Ma, Jian Chen, Xiwen Lin, Daoqin Zhang, Zhen Li, Chunwei Zheng, Yanmin Feng, Xingxu Huang, Chunsheng Han

**Affiliations:** 1 State Key Laboratory of Stem Cell and Reproductive Biology, Institute of Zoology, Chinese Academy of Sciences, Beijing 100101, China; 2 University of Chinese Academy of Sciences, Beijing 100049, China; 3 State Key Laboratory of Reproductive Medicine, Department of Histology and Embryology, Nanjing Medical University, Nanjing 210029, China; 4 School of Life Science and Technology, Shanghai Tech University, Shanghai 201210, China

## Abstract

ZMYM3, a member of the MYM-type zinc finger protein family and a component of a LSD1-containing transcription repressor complex, is predominantly expressed in the mouse brain and testis. Here, we show that ZMYM3 in the mouse testis is expressed in somatic cells and germ cells until pachytene spermatocytes. Knockout (KO) of *Zmym3* in mice using the CRISPR-Cas9 system resulted in adult male infertility. Spermatogenesis of the KO mice was arrested at the metaphase of the first meiotic division (MI). ZMYM3 co-immunoprecipitated with LSD1 in spermatogonial stem cells, but its KO did not change the levels of LSD1 or H3K4me1/2 or H3K9me2. However, *Zmym3* KO resulted in elevated numbers of apoptotic germ cells and of MI spermatocytes that are positive for BUB3, which is a key player in spindle assembly checkpoint. *Zmym3* KO also resulted in up-regulated expression of meiotic genes in spermatogonia. These results show that ZMYM3 has an essential role in metaphase to anaphase transition during mouse spermatogenesis by regulating the expression of diverse families of genes.

Mammalian spermatogenesis is a unique cellular developmental process that is intricately regulated by extrinsic and intrinsic factors. Spermatogonial stem cells (SSCs) at the very beginning of spermatogenesis have to make decisions either to undergo self-renewal or to initiate differentiation, which eventually leads to meiosis and sperm production. The mechanism by which such a decision is made remains poorly understood despite that several key factors have been identified. For example, glial cell-derived neurotrophic factor (GDNF) promotes SSC self-renewal and inhibits their differentiation, whereas retinoic acid (RA) acts in an opposite manner.^1, 2^ The lengthy differentiation of spermatogonia, tightly coupled with active mitotic divisions, results in drastic amplification of spermatogenic population and an orderly gene expression change that is essential for meiosis as well as post-meiotic development. Abnormal gene expression during spermatogonial differentiation leads to either a spermatogenic arrest at a pre-meiotic stage or a precocious entry of meiosis.^3, 4^

Oatley *et al.*^5^ identified GDNF-regulated genes using microarray analysis on cultured mouse SSCs and showed that several such genes were essential for SSC self-renewal. Among their down-regulated gene list, we identified a gene named *Zmym3*. An early study reported that *Zmym3* mRNA was most abundant in adult testis and brain among eight examined organs and was alternatively spliced in a development- and tissue-specific manner and that protein sequences of *Zmym3* are evolutionarily conserved from the arthropods to humans with several highly conserved protein motifs.^6^ ZMYM3 and one of its paralog ZMYM2 each contain nine similar zinc fingers. In humans, chromosome translocations near these two genes have been linked to myeloproliferative syndromes and X-linked mental retardation, respectively.^7, 8^ Therefore, the zinc fingers in these two proteins are named MYM (myeloproliferative and mental retardation)-type zinc fingers. Based on the current NCBI HomoloGene database, the human and mouse genomes encode six MYM-type ZFPs, which are ZMYM1, ZMYM2, ZMYM3, ZMYM4, ZMYM5, and ZMYM6. The other motifs of ZMYM3, which are potentially important for its function, include nuclear localization signal, SH3-binding motif and tyrosine phosphorylation sites, suggesting that the function of this protein is highly regulated.

Interestingly, both ZMYM3 and ZMYM2 were identified in a LSD1-containing complex isolated from Hela cells.^9^ LSD1 is the first identified histone demethylase,^10^ and has since been shown to have essential roles in many biological processes.^11^ Specific deletion of *Lsd1* in mouse oocytes results in female infertility due to precocious meiotic resumption, spindle and chromosomal abnormalities, and disrupted gene expression during oogenesis.^12^ Similarly, conditional deletion of *Lsd1* in mouse spermatogonia leads to male infertility as a result of abnormal histone modification and gene expression in spermatogonia followed by a complete loss of germ cells in adult males.^13^ The function of ZMYM proteins has not been well understood except for that ZMYM2 is known to stabilize the LSD1–CoREST–HDAC1 transcriptional co-repressor complex on chromatin through its MYM-type zinc fingers.^14^

In this study, we report that mouse *Zmym3* in cultured SSCs is regulated by GDNF and RA in opposite ways and expresses two major protein isoforms. During spermatogenesis, ZMYM3 is present in germ cells until pachytene spermatocytes (pacSC). *Zmym3*gene KO in mice by using the CRISPR-Cas9 system results in infertility in adult male mice. Spermatogenesis of the KO mice exhibits a major arrest at the metaphase of meiosis I (MI). The longer protein isoform but not the short one interacts with LSD1. However, the *Zmym3* gene KO has no effect on the LSD1 protein level and on total abundances of H3K4me1/2 or H3K9me2. The spindle body formation is normal but more apoptotic and BUB3^+^ MI cells are observed in KO mice. RNA sequencing analysis of cultured SSCs and isolated spermatocytes shows that many genes are expressed aberrantly. These results shows that ZMYM3, a highly conserved ZMYM-type LSD1 interacting protein, has an essential role in spermatogenesis in an organ-specific manner.

## Results

### *Zmym3* is down-regulated by GDNF, up-regulated by RA, and expressed in germ cells until pacSC

**Figure 1:**
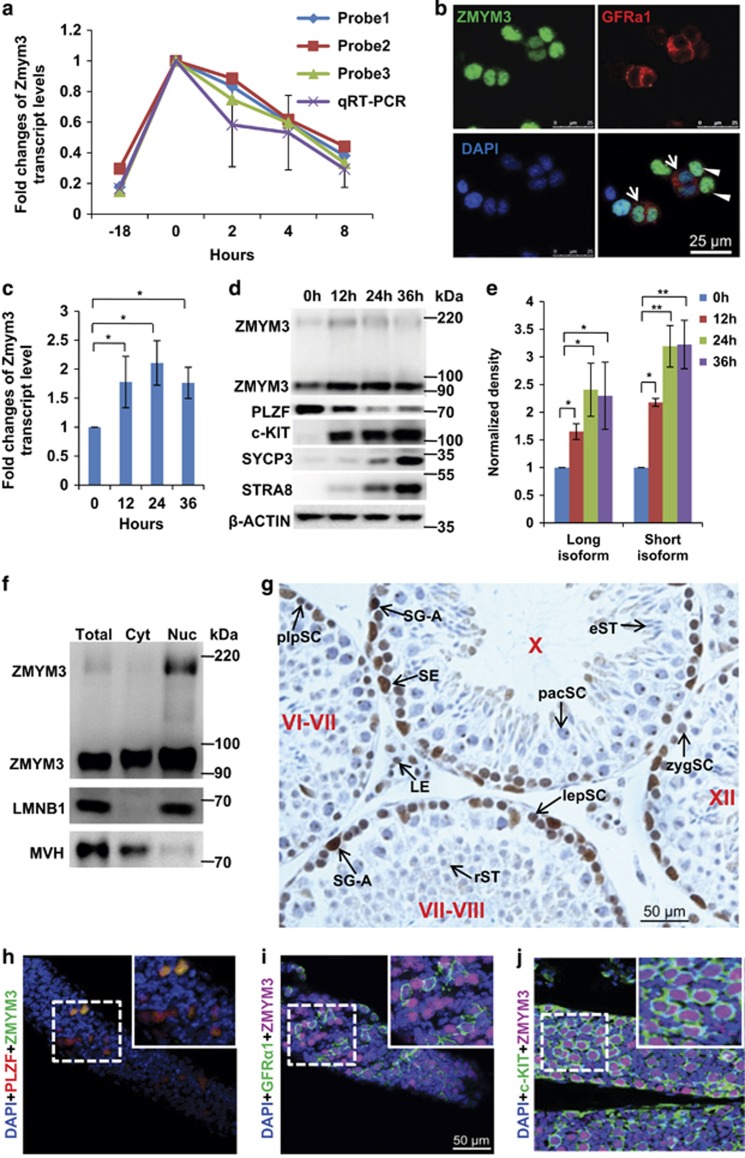
Expression of ZMYM3 in cultured mouse spermatogonial stem cells and in cells of the mouse testis. (**a**) *Zmym3* mRNAs in cultured mouse SSCs are down-regulated by GDNF (*n*=3). (**b**) Expression of ZMYM3 in cultured SSCs. Note that the signal of ZMYM3 is stronger in SSCs which are weaker in GFR*α*1 staining (arrow heads) but weaker in SSCs which are stronger in GFR*α*1 staining (arrows). (**c–e**) *Zmym3* mRNAs and proteins in cultured SSCs are up-regulated by RA as determined by qRT-PCR (**c**) (*n*=3) and Western blot assays (**d**) (*n*=4). (**e**) is the quantitative densitometry of the Western blot result in (**d**). (**f**) Western blot of ZMYM3 in cytoplasm and nuclear fractions of cultured SSCs. Total: total cell lysates; Cyt: cytoplasmic fraction; Nuc: nuclear fraction; MVH: Mouse Vasa Homolog, a germ cell specific marker and a cytoplasmic protein; LMNB1: a component of the nuclear lamina and a nuclear protein. (**g**) Immunohistochemical analysis of ZMYM3 distribution in different testicular cells of adult mice. Note that a small dot in the spermatocyte most likely representing the sex body was positively immunostained. However, it turned out to be a nonspecific signal because it was also observed in *Zmym3* KO mice (Figure 2e). Roman numerals indicate the stage of spermatogenesis. SG-A, spermatogonia type A; plpSC, preleptotene spermatocytes; lepSC, leptotene spermatocytes; zygSC, zygotene spermatocytes; pacSC, pachytene spermatocytes; rST, round spermatids; eST, elongating spermatids; SE, Sertoli cells; LE, Leydig cells. (**h**–**j**) Whole-mount co-immunostaining of ZMYM3 with PLZF and GFR*α*1 on seminiferous tubules from P6 mice and c-KIT on those from P8 mice

Based on a microarray data set reported by Oatley *et al.,*^5^ we found that *Zmym3* mRNAs were down-regulated by GDNF, which was consistently supported by results from three different probe sets on the microarray. Taking advantage of their experiment design, we confirmed this observation by using our own cultured SSCs and quantitative RT-PCR (qRT-PCR) assays (Figure 1a). The presence of ZMYM3 protein in cultured SSCs was detected by immunostaining using a commercially available polyclonal antibody (Figure 1b, Supplementary Figure S1 and S2a). The identity of the ZMYM3^+^ SSCs was confirmed by the expression of GDNF receptor subunit GFR*α*1 (Figure 1b). Interestingly, we noticed that the signals of ZMYM3 and GFR*α*1 were negatively correlated. As GFR*α*1 expression is higher in actual stem cells than in potential stem cells that have undergone slight differentiation,^15^ we suspected that the expression of *Zmym3* might also be regulated by RA. Indeed, *Zmym3* expression was up-regulated by RA at both the mRNA and protein levels in a time-dependent manner (Figure 1c–e).

On Western blots, we saw two ZMYM3 bands (~200 and 95 kDa), which represent isoforms probably translated from alternatively spliced mRNAs as many verified and predicted alternatively spliced mRNAs were reported by a previous study and the NCBI Gene database.^6^ Because the sizes of both bands on Western blots are larger than the predicted masses of the corresponding isoforms (Supplementary Figure S2b), they are most likely post-translationally modified. We further examined the subcellular localization of the two isoforms using cytoplasmic and nuclear extracts from SSCs and found that the larger form was predominantly localized to the nucleus, whereas the smaller one was detected in both the cytoplasm and the nucleus (Figure 1f).

We next investigated *Zmym3*mRNA and protein expression in testicular cells and other organs of adult mice. All results of RNA sequencing (RNA-seq),^16^ qRT-PCR and Western blotting showed that ZMYM3 was ubiquitously expressed in multiple organs, and was the most abundant in gonads and brain (Supplementary Figure S2c–g). In the testis, ZMYM3 was expressed in both germ cells and somatic cells such as Sertoli cells and interstitial cells, and among germ cells, ZMYM3 was expressed in spermatogonia and early spermatocytes such as preleptotene, leptotene, and zygotene spermatocytes (plpSC, lepSC, and zygSC) but not in late spermatocytes such as pacSC or spermatids (Figure 1g), consistent with the RNA-seq^17^ and qRT-PCR results (Supplementary Figure S2c–d). Whole-mount co-immunostaining of ZMYM3 with GFR*α*1, PLZF (markers for undifferentiated spermatogonia), and c-KIT (a marker for differentiating spermatogonia and plpSC) on the seminiferous tubules showed that ZMYM3 was expressed in all stage spermatogonia with a higher level in c-KIT^+^ differentiating spermatogonia and probably the plpSC (Figure 1h–j).

### *Zmym3* KO in mice results in adult male infertility and arrests spermatogenesis at MI

**Figure 2:**
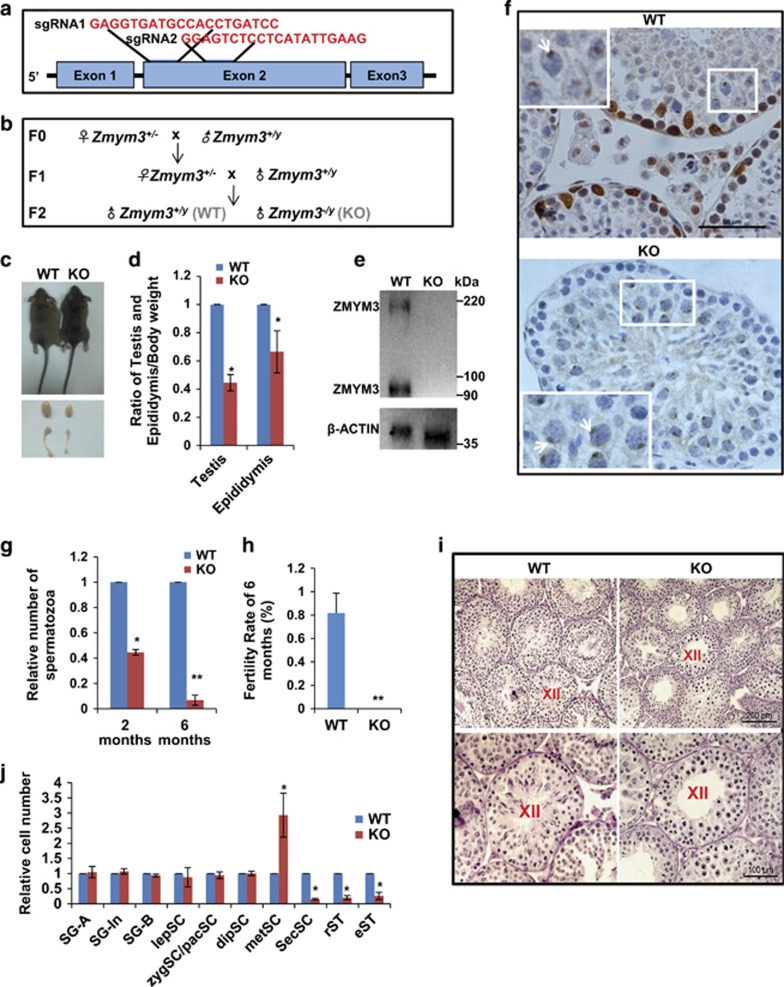
Generation and phenotypic analysis of *Zmym3* KO mice. (**a**) A schematic diagram of sgRNAs targeting the *Zmym3* loci. (**b**) Breeding strategy to generate *Zmym3* KO male mice. To exclude any unexpected phenotypes due to potential chimeric DNA deletions, we used two female founders (founder No. 1 and 2 in Supplementary Figure S3a) to cross with wild-type males for two generations to produce F2 homozygous males for in-depth phenotypic analysis. (**c**) Comparisons of the body, testis, and epididymis sizes of WT and KO mice at ages of 6 months. (**d**) Quantitative comparisons of the testis and epididymides weight betweenWT and KO mice at ages of 2 months. (**e**) Western blot analysis of ZMYM3 in WT and KO mice using total testicular cell lysates. (**f**) The absence of *Zmym3* in KO mice shown by ZMYM3 immunostaining. Note the nonspecific immunostaining of the sex body in both WT and KO mice indicated by the white-boxed insets. (**g**) Sperm count comparison between WT and KO mice at ages of 2 months and 6 months. (**h**) Fertility rates of WT and KO mice at ages of 6 months. (**i**) Histological analysis of testis sections from WT and KO mice at ages of 6 months. (**j**) Quantification of spermatogenic cell types in WT and KO adult mice based on the images shown in Supplementary Figure S4. For each cell type, at least a total of nine tubules from three mice were counted. The average numbers of cells per tubule were converted to ratios and compared between WT and KO mice (*n*=9)

We next generated *Zmym3* KO mice by injecting into fertilized eggs Cas9 mRNAs and two sgRNAs targeting the second exon of the gene (Figure 2a). Four female founder mice were generated with small deletions in the expected genomic region, resulting in premature termination of translation owing to frame shifts (Supplementary Figure S3a). We next identified the most probable off-target sites for each gRNA by bioinformatic predictions, and found no mutations on these sites by sequencing. Adult KO males older than 6 months did not show any apparent abnormal appearance or behavior and mated with females normally as vaginal plugs were regularly seen. However, the KO testes and epididymides were significantly smaller than the WT ones (Figure 2c and d). Western blotting and immunohistochemical results both confirmed the complete KO of ZMYM3 in the KO testis (Figure 2e and f). The KO males started to reduce their testis sizes, sperm counts, and fertilities from 2 months after birth (Supplementary Figure S3b). Interestingly, the smaller number of spermatids produced in young animals differentiated to spermatozoa normally, and showed no obvious apoptosis during their postmeiotic development (Supplementary Figure S3c). Moreover, spermatozoa produced from the KO mice were also morphologically normal (Supplementary Figure S3d). The KO mice became infertile when tested at 6 months after birth (Figure 2g and h). A close look at the PAS-stained testis sections of the KO mice showed that their spermatogenesis was mainly arrested at MI (Figure 2i). The numbers of round spermatids, elongating spermatids, as well as sperms were all significantly reduced (Figure 2j, Supplementary Figure S4).

### *Zmym3* KO mice undergo spermatogenesis normally until MI

**Figure 3:**
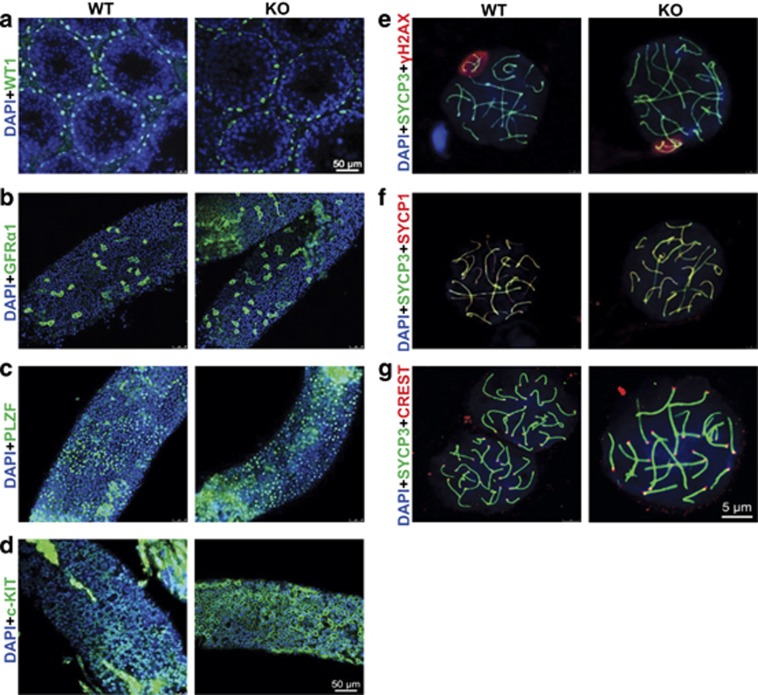
Phenotypic evaluation of testicular cells in 6-month *Zmym3* KO mice. (**a**) Immunofluorescent images of cross sections of WT and KO testes with the anti-WT1 antibodies and stained with DAPI to show nuclei. (**b**–**d**) Immunofluorescent images of whole-mount immunostained seminiferous tubules using anti-GFR*α*1, anti-PLZF and anti-c-KIT antibodies. (**e**–**g**) Immunofluorescent images of chromosome spread of spermatocytes for SYCP3 with *γ*H2AX, SYCP1, and CREST

As ZMYM3 is also expressed in Sertoli cells, we first examined the immunostaining of the Sertoli cell marker Wilms Tumor 1 (WT1),^18^ but found no difference between WT and KO mice in terms of its localization and the number of WT1^+^ cells. Similarly, no difference was found for GFR*α*1, PLZF, and c-KIT. These results indicate that the development and probably the function of both somatic cells and pre-meiotic germ cells are not changed by *Zmym3* KO (Figure 3a–d). When meiosis is initiated in lepSC, these cells undergo DNA double-strand breaks (DSBs) and chromosomal synapsis, and the progression of these two processes can be monitored by the immunostaining patterns of proteins such as *γ*H2AX, SYCP3, SYCP1, and CREST. In pacSC of both WT and KO mice, the DSBs had all been repaired and autosomes fully synapsed as shown by localization of *γ*H2AX signals to the partially synapsed sex chromosomes and by the noodle-like bright smooth staining of SYCP3 and SYCP1 (Figure 3e–g, Supplementary Figure S5). Together, these data suggest that the mitosis and meiosis phases before MI in *Zmym3* KO mice are normal.

### *Zmym3* KO does not change the *in vitro* proliferation and meiosis initiation of SSCs

**Figure 4:**
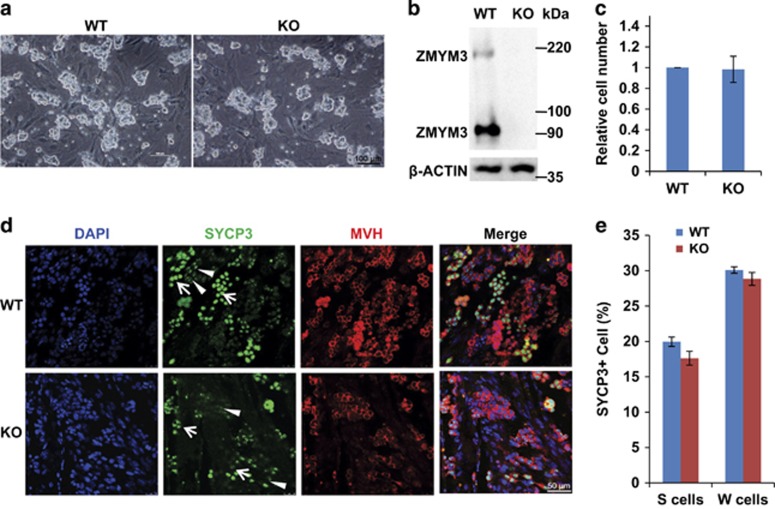
Characterization of cultured SSCs from *Zmym3* KO mice. (**a**) Bright-field images of cultured WT and KO SSCs. (**b**) Western blot of ZMYM3 protein in WT and KO SSCs. (**c**) Quantitative evaluation of proliferations of WT and KO SSCs. Equal numbers of WT and KO SSCs were seeded on MEF feeder. Cell numbers are counted 5 days later and normalized by the number of WT SSCs. (**d**) Immunostaining of SYCP3^+^ cells induced from WT and KO SSCs. Germ cell marker MVH was co-immunostained. S-cells (arrows); W-cells (arrow heads). (**e**) Quantitative comparisons in the percentages of S-cells and W-cells among all induced SYCP3^+^ cells between WT and KO SSCs. Equal numbers of WT and KO SSCs were planted on Sertoli cells. The images were taken on day 6 of induction represented by (**d**). The ratios of S- and W-cells were calculated based on the results from three independent assays (*n*=3)

Several SSC lines developed from the F2 KO mice and their WT littermates were successfully established, indicating that *Zmym3* KO might not impair the *in vitro* proliferation of SSCs (Figure 4a). The KO of *Zmym3* in these cultured SSCs was confirmed by Western blot (Figure 4b). The proliferation rate of these KO cells was quantitatively compared with that of WT SSCs and no significant difference was found (Figure 4c). We next cultured WT and KO SSCs on Sertoli cells and induced them by RA to initiate meiosis.^19^ Five days after RA treatment, SYCP3^+^ cells were observed. The weakly and strongly stained cells represented plpSC and lepSC/zygSC, and were named W-cells and S-cells, respectively, for convenience (Figure 4d). Again, no difference was observed for the percentages of either W-cells or S-cells between KO and WT SSCs (Figure 4e). Taken together, these results indicated that neither the proliferation nor the meiosis initiation of cultured SSCs was damaged by *Zmym3* KO, and the *in vitro* results were consistent with the *in vivo* observations.

### *Zmym3* KO has no effect on the protein levels of LSD1, H3K4me1/2, and H3K9me2

**Figure 5:**
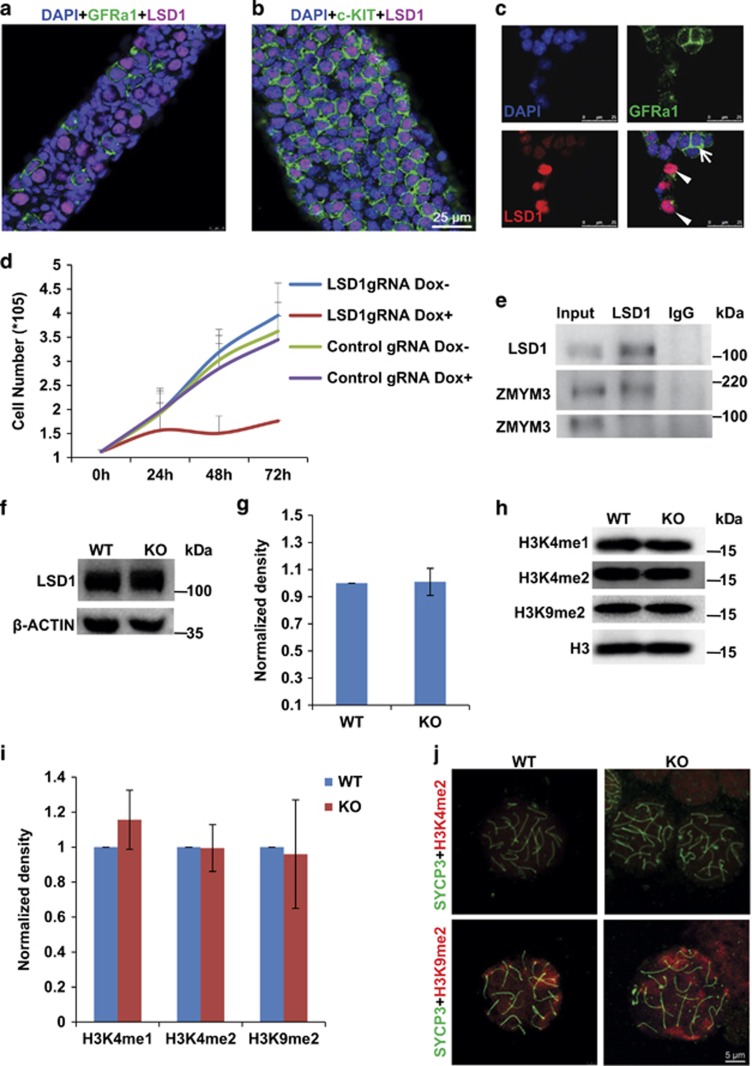
Relationship between ZMYM3 and LSD1 and epigenetic modifications. (**a**–**b**) LSD1 is expressed in c-KIT^+^ cells at a higher level than in GFR*α*1^+^ cells. Whole-mount co-immunostainings of LSD1 with GFR*α*1 and c-KIT were conducted on seminiferous tubules from P6 and P8 mice, respectively. (**c**) Co-immunostaining of LSD1 with GFR*α*1 in cultured SSCs. Note the inverse relationship in the signals of LSD1 and GFR*α*1. The signal of LSD1 is stronger in SSCs which are weaker in GFR*α*1 staining (arrow heads) but weaker in SSCs which are stronger in GFR*α*1staining (arrows). (**d**) KO of *Lsd1* in cultured inducible Cas9-SSCs with Doxycyclin suppresses the proliferation of SSCs (n =3). (**e**) The larger isoform but not the smaller one of ZMYM3 co-immunoprecipitates with LSD1 using an antibody against LSD1. (**f**–**g**) Western blot of LSD1 expression in WT and KO SSCs (*n*=3). (**g**) is the quantitative densitometry of the Western blot result in (**f**). (**h**–**i**) Western blot of H3K4me1/2 and H3K9me2 in WT and KO SSCs (*n*=3). (**i**) is the quantitative densitometry of the Western blot result in (**h**). (**j**) Immunostaining analysis of H3K4me2 and H3K9me2 in spermatocyte spreads of WT and KO mice

A previous study showed that LSD1 was expressed at a much higher level in mouse testis than in other organs, such as brain, lung, liver, heart, and was detected in all types of spermatogenic cells.^20^ Using whole-mount immunostaining, we found that LSD1, similar to ZMYM3, was more abundantly expressed in c-KIT^+^ cells than in GFR*α*1^+^ cells (Figure 5a and b). LSD1 also exhibit a similar expression pattern as ZMYM3 in cultured SSCs as indicated by its co-immunostaining with GFR*α*1 (Figure 5c). We showed that *Lsd1* KO using an inducible Cas9-SSC line, which was established in our lab recently,^21^ reduced the proliferation of SSCs significantly (Figure 5d), consistent with its essential role in spermatogenesis.^13^ Co-immunoprecipitation assay showed that an LSD1 polyclonal antibody pulled down both LSD1 and the larger but not the smaller form of ZMYM3 (Figure 5e). Despite the interaction of ZMYM3 and LSD1 in SSCs, we found that LSD1 was expressed in cultured WT and KO SSCs at similar levels based on the Western blot results (Figure 5f–g). Moreover, the global levels of H3K4me1/2 and H3K9me2 were not changed by *Zmym3* KO (Figure 5h–i). No apparent difference was also observed for H3K4me2 and H3K9me2 immunostainings in pacSC isolated from WT and KO mice (Figure 5j). These results showed that ZMYM3 KO had no apparent effect on the global levels of LSD1 and the examined histone modifications both *in vitro* and *in vivo.*

### *Zmym3* KO causes MI arrest in a SAC-dependent manner

**Figure 6:**
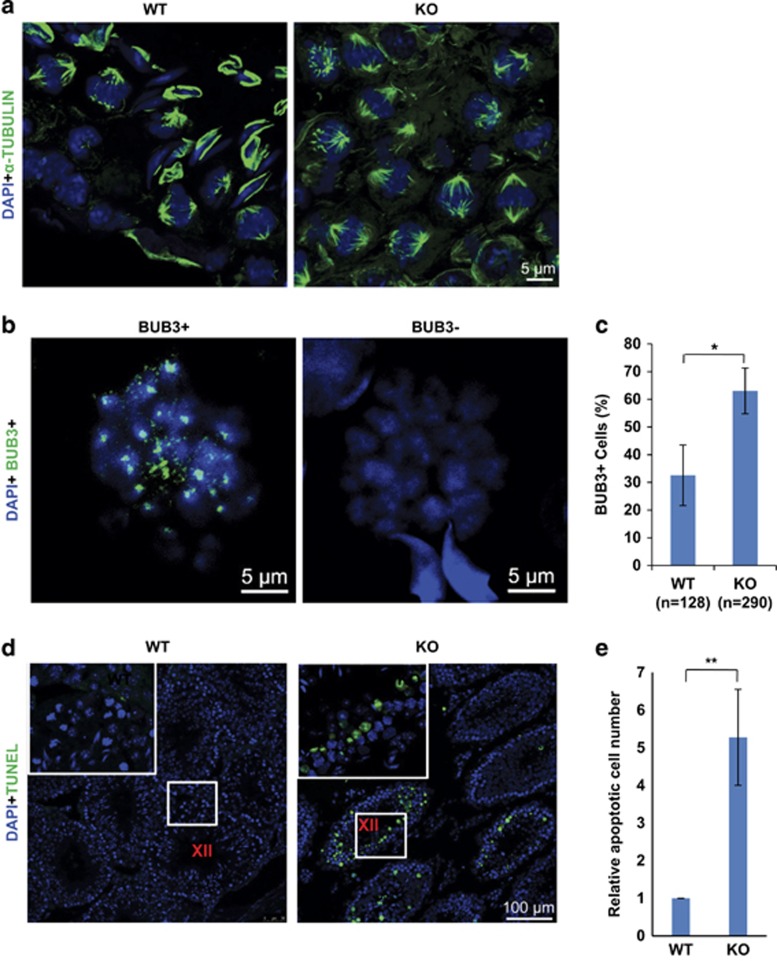
*Zmym3* KO causes MI arrest through a SAC-dependent pathway. (**a**) Immunofluorescent images of cross sections of 6-month mice testes with the anti-*α*-TUBULIN antibodies. (**b**) Immunofluorescent images of chromosome spread of spermatocytes for BUB3. (**c**) Quantitative results of the percentages of BUB3^+^ cells among MI spermatocytes from WT and KO mice. The ratios of BUB3^+^ cells were calculated based on the results from 128 and 290 cells from four WT and four KO mice. (**d**) Cell apoptosis assay by TUNEL staining of testis sections from WT and KO mice. (**e**) Quantitative results of apoptotic cells among MI spermatocytes

For both mitosis and meiosis, cells use spindle assembly checkpoint (SAC) to ensure the fidelity of chromosome segregation.^22^ Proteins involved in SAC include BUB1, BUBR1, BUB3, and MAD2.^23^ As *Zmym3* knockout (KO) caused an accumulation of MI spermatocytes, we examined whether any of these proteins was abnormally localized in meiotic cells of the KO mice. *Zmym3* KO did not affect spindle assembly as revealed by the *α*-TUBULIN staining (Figure 6a). However, the number of BUB3^+^ MI spermatocytes in *Zmym3* KO mice was about two-fold more than that in WT mice (Figure 6b and c). Moreover, TUNEL assays indicated that significantly more MI spermatocytes in KO mice underwent apoptosis than in WT mice (Figure 6d and e). These results showed that *Zmym3* might regulate metaphase–anaphase transition through a SAC-dependent pathway.

### *Zmym3* KO disrupts mRNA expression of genes involved in meiosis and post-meiotic development of germ cells

**Figure 7:**
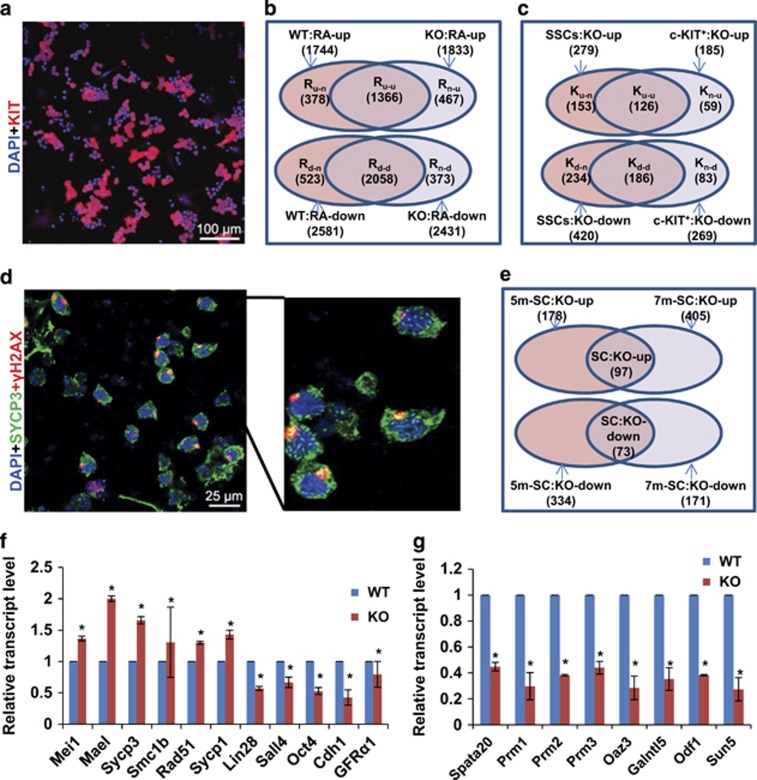
RNA-Seq analysis of the effect of *Zmym3* KO on gene expression in cultured SSCs and isolated spermatocytes. (**a**) c-KIT^+^ cells were induced from both WT and KO SSCs by 100 nM RA 36 h after RA treatment. Only result for WT SSCs is shown. (**b**) Gene sets up- or down-regulated by RA in WT and KO SSCs. (**c**) Gene sets up- or down-regulated by *Zmym3* KO in SSCs and induced c-KIT^+^ cells. (**d**) Immunostaining of SYPC3 and *γ*H2AX on spermatocytes isolated from WT or KO mice to show that higher than 80% of the cells were double positively stained. Only the result for WT mice is shown. (**e**) Gene sets up- or down-regulated by *Zmym3* in isolated spermatocytes from 5- and 7-month mice. (**f**–**g**) qRT-PCR evaluation of the expression of genes identified by RNA-seq analysis

To elucidate the molecular bases for the infertile phenotype of *Zmym3* KO mice at the mRNA expression level, we first carried out RNA-seq analysis on cultured SSCs and c-KIT^+^ pre-meiotic cells induced from SSCs by RA treatment for both WT and KO mice (Supplementary Table S1). To acquire c-KIT^+^ cells, feeder-free SSC cultures were induced by 100 nM RA for 36 h and 90% of the cells became c-KIT^+^ (Figure 7a). We first found 1744 and 2581 genes to be either up- or down-regulated by RA in WT SSCs (Figure 7b). Interestingly, 467 novel RA-upregulated genes (set R_n-u_ in Figure 7b) were identified after *Zmym3* was knocked out, and this gene set was found to be significantly enriched with zinc finger family transcription factors (Table 1). Moreover, we found that many genes involved in meiotic cell cycle and spermatogenesis such as *Sycp1*, *Sycp2*, *Mov10l1, Rnf17*, *Stag1,* and *Smc* were precociously expressed when *Zmym3*was knocked out (set R_u-n_ in Figure 7b, sets K_u-n_, K_u-u_, K_n-u_ in Figure 7c). The up-regulation of some of these in KO SSCs was confirmed with qRT-PCR using independent samples (Figure 7f).

We found that smaller numbers of genes changed their expression in response to *Zmym3* KO in SSCs and c-KIT^+^ cells compared with RA-regulated genes. Some genes involved in the proliferation regulation of SSCs and undifferentiated spermatogonia such as *Lin28*, *Sall4*, *Oct4*, *Cdh1,* and *Gfrα1* were also in the KO-down set, and their expression changes caused by *Zmym3* KO were also confirmed by qRT-PCR (Figure 7f). These observations suggest that *Zmym3* KO enhances the expression of genes involved in spermatogonia differentiation and meiosis while suppresses genes that maintain the undifferentiated states of spermatogonia.

We next conducted RNA-seq analysis on spermatocytes directly isolated from mouse testes by sorting out tetraploid cells, which were mainly SYCP3 and γH2AX double positive (Figure 7d). We found 97 up-regulated and 73 down-regulated genes in spermatocytes of both 5- and 7-months (Figure 7e). We checked the expression of 24 genes involved in SAC but found they were not dysregulated by *Zmym3* (Supplementary Figure S6b). The down-regulated set was enriched with several GO terms such as ‘spermatogenesis’, which included genes such as *Prm1*, *Prm2*, *Prm3*, *Klhl10*, *Odf1*, *Chd5*, *Sun5*, *Ccdc63*, *Oaz3*, *Spata20*, *Galntl5*, *Atp1a4*, *Acsbg2*, which either have essential roles or are highly/specifically expressed in spermatids, the expression of which were also confirmed by qRT-PCR (Figure 7g). These results indicated that *Zmym3* KO disrupted the expression of some key genes involved in postmeiotic development of spermatogenesis.

## Discussion

We report in this study that *Zmym3*, a gene that initially came to people’s attention for its potential roles in X-linked mental retardation and epigenetic regulation, has an essential role in mouse spermatogenesis. *Zmym3* KO mice have no other apparent abnormalities including mating behavior despite that the gene is highly expressed in the brain, but arrests spermatogenesis at MI through a SAC-dependent pathway. Therefore, we have identified an evolutionarily conserved gene that has a specific role in promoting meiosis progression during spermatogenesis. The female KO mice seemed to be fertile as they gave birth to mutant offspring, but this question remains open until the fertility of homozygous female KO mice is carefully evaluated in the future.

Given that ZMYM3 is expressed in both somatic cells and germ cells, we are not sure whether ZMYM3 in somatic cells has a role in spermatogenesis based on the results in the present study. Despite that the function of Sertoli cells and the androgen-producing Leydig cells both seem to be normal based on the immunostaining of WT1 and the normal mating behavior of the KO mice, that the KO mice do not loss their fertility completely until 6 months after birth suggests that this phenotype may also be related to the senescence of somatic cells. This question can be addressed in the future by transplanting WT SSCs into the testes of infertile KO mice and checking whether spermatogenesis can be re-established.

Given that ZMYM3 is expressed in all spermatogenic cells before meiosis initiation, it is surprising that no apparent defects are observed earlier than in MI spermatocytes. One explanation is that subtle defects do occur in these cells but cannot be easily detected, and they accumulate to a point of no-return whereby spermatogenesis arrests at MI. This is supported by the RNA-seq results, which show that many genes express abnormally in cultured KO cells. Particularly, some genes that are involved in meiotic processes such as synapsis are up-regulated in KO SSCs, suggesting that the KO spermatogonia might initiate meiosis precociously. Alternatively, these cells are indeed normal because the lost function of ZMYM3 in KO germ cells is compensated by other similar proteins. This explanation is supported by the observation that quite a few zinc finger protein genes were up-regulated by RA in germ cells only when *Zmym3* is knocked out. It is more surprising that no recognizable genes involved in meiosis are dysregulated in the KO spermatocytes. Such a discrepancy between *Zmym3* expression pattern and the time point when its function was clearly revealed by gene KO suggests the presence of a complex functional regulatory pathway, which may consist of multi-step protein–protein interactions. Indeed, we have identified many ZMYM3-interacting partners including some transcription co-factors and a protein involved in sumoylation using yeast-two-hybrid methods (data not shown). The detection of up-regulated expression of haploid genes in KO spermatocytes should be cautioned. Despite that the isolation of spermatocytes was carried out based on both their tetraploid feature and their large size, minor contamination of secondary spermatocytes is still possible. As the genes such as Prm1/2/3 are highly expressed, its expression can be readily detected even if the contamination is minor, and the difference in their expression between WT and KO mice can also be detected as the KO testes lack secondary spermatocytes.

ZMYM3 has been reported to be associated with epigenetic modifying enzymes such as LSD1, HDAC1/2 by several studies. Two isoforms of ZMYM3 are present in SSCs and the larger form but not the short one co-immunoprecipitates with LSD1. Interestingly, *Zmym3* KO does not change the expression level of LSD1, H3K4me1/2, and H3K9me2. However, this does not exclude the possibility that epigenetic modifications on certain genomic regions are disrupted but not detected by Western blotting or immunocytochemical assays. It is important to observe that *Zmym3* KO results in MI arrest related to SAC, which has been well studied in oogenesis.^24^ MI-arrested spermatocytes triggered by SAC were eliminated through apoptosis, a male-specific event.^25^ A Y chromosome-located gene named *Zfy2*, which also encodes a zinc finger protein, has been reported to be essential and sufficient for removing the apoptotic MI-arrested spermatocytes.^26^ Therefore, ZMYM3 might represent a novel sex-specific player in this pathway if the female KO mice are indeed fertile.

A recent study showed that ZMYM3 in HEK293T cells had an essential role in DNA damage repair through the homologous recombination pathway by interacting with both histone and DNA components of the nucleosome.^27^ Moreover, another study reported that SAC was a major gatekeeper preventing the progression of oocytes harboring DNA damage.^28^ Based on these studies, it is tempting to propose that ZYMY3 is also involved in DSB repair during meiosis of spermatocytes, the failure of which activates SAC and causes apoptotic elimination of damaged cells. However, we were unable to acquire evidences for this hypothesis. The originally observed immunostaining signal in sex body was most likely nonspecific because it was also detected in KO testes; the immunostaining patterns of *γ*H2AX in pacSC of both WT and KO testes were not different and indicated DSBs were repaired normally. Despite of these observations, we still cannot reject this hypothesis confidently as subtle DNA damages may exist but escape from detection owing to the low resolution of the methods used in the present study. In the future, we will continue to test this hypothesis by using more sensitive techniques to detect regional epigenetic modifications as well as DNA damages.

## Materials and methods

### Mice

All animal protocols were approved by the Animal Care and Use Committee of the Model Animal Research Center, the host for the National Resource Center for Mutant Mice in China, Nanjing University and the Animal Care and Use Committee of the Institute of Zoology, Chinese Academy of Science. *In vitro* transcription of Cas9 mRNAs from pST1374-Cas9-N-NLS-flag-linker and sgRNAs from pUC57-sgRNA expression vectors was performed as described previously.^29^ The sequences of sgRNA oligos are listed in Supplementary Table S2. Cas9 mRNA/sgRNA injection to zygotes obtained by mating of CBA males with superovulated C57BL/6J females was also performed as described previously.^30^ Female mice with a frame shift and premature termination at an out-of-frame stop codon were chosen as founder animals. Pregnancies were established when female *Zmym3*^+/−^ mice were mated to wild-type males.

### Culture, differentiation, and gene KO of mouse SSCs

Mouse SSCs were obtained from the testes of pup (5–7 dpp) or adult mice by following procedures previously reported.^31^ The induction of c-KIT^+^ cells from SSCs were conducted by following our protocol recently pulished.^19^ The KO of *Lsd1* in SSCs was performed using an inducible Cas9-SSC line (iCas9-SSC) established recently.^21^ Sequences for sgRNAs targeting *Lsd1* were included in Supplementary Table S2.

### RNA extraction, qRT-PCR, and RNA sequencing

The isolation of spermatocytes was carried out by first sorting out tetraploid cells from total testicular cells and then selecting spermatocytes based on their forward scatter and side scatter features in FACS analysis (Supplementary Figure S6a). The purity of spermatocytes was higher than 80% as shown by the immunostainings of SYCP3 and *γ*H2AX. Total RNA from mouse testis cells and mSSCs was extracted using Trizol (Invitrogen, Carlsbad, CA, USA) according to the standard protocol. After reverse transcription of purified RNA performed using Reverse Transcription System (G3250, Promega, USA) according to the manufacturer’s protocols, qPCRs were conducted with UltraSYBR Mixture (CW0956, CoWin Biotech, Beijing, China) by following the manufacturer’s instructions on a LightCycler 480 platform (Roche Diagnostics, Basel, Switzerland). Data were acquired in biological triplicates. Relative gene expression was calculated based on ΔΔCt method using *β*-Actin as an internal control. All primer sequences of selected genes were listed in Supplementary Table S2. Prior to sequencing, the total RNA was subject to DNase treatment to eliminate genomic DNA contaminants. The quality of the RNA samples was assessed by agarose gel electrophoresis and RT-PCR detection of the expressions of selected genes. RNA samples were prepared for sequencing on the Illumina HiSeq 2000 platform (Illumina, San Diego, CA, USA). Data analysis was performed as previously described.^17^ Differentially expressed genes were identified if their *q*-values reported by the Cuffdiff software were <0.01 unless otherwise stated. GO term enrichment analyses were performed using the online DAVID program. A GO term was considered to be significantly enriched if the enrichment false discovery rate Bejamini is <0.05.

### Data analysis and statistics

Statistical analyses were performed using *t*-test. Results are presented as mean±S.D. In all figures, * and ** denote that *P*<0.05 and 0.01, respectively. All experiments were independently repeated at least three times.

## Figures and Tables

**Table 1:** Differentially expressed genes in cultured Zmym3 WT and KO SSCs and isolated spermatocytes by RNA-Seq analysis

**Subset**	**Enriched GO terms**	**Genes**
Rn-u (*q*<0.01)	Regulation of transcription, DNA-templated	ZFP12, ZFP40, MAF1, ZKSCAN3, ZFP788, ZFP786, MAP3K7, EPC1, MDFIC, RNF38, ZFP687, ZFP503, ZFAT, ZFP882, INSR, NFX1, ZFP518A, ZFP422, ZFP423, SATB2, KHDRBS3, RBL2, ZFX, ZHX1, ZFP629, ZFP128, ZFP592, ZFP827, ZFP120, ZFP280C, IGSF1, NCOA5, PRDM5, ZFP697, MAPK8, ZFPM1, ZFP516, ZFP229, ZFP511, ZFP369, ZSCAN12, MEAF6, ZFP715, ERBB4, ZFP612, ZFP398, ZFP318, ZFP113, ZFP319, AI987944, ZFP768, ZFP316, ARNT, ZFP317, MED12L, MYCBP2, HIC2, ZFP956, RB1CC1, ZFP410, TRP53INP2, NKX3-1, MLLT1, ZFP217, ZFP810, SLC30A9, CHD5, ZFP251, ZFP382, TGFBR1, KLF11, KCTD1, ZBTB41, ZFP445, ZFP709, ZFP746, ZFP809, SP3, ZFP282, ZFP488, HOXB6, ZFP800, ZFP536, HDAC8, KLF4
Ru-n (*q*<0.01)	Cell cycle	ARHGEF2, STOX1, SYCP2, MCM3, SYCP1, SMC2, LATS2, SPDYA, RIF1, PMP22, UBE2S, HELLS, STAG1
KO-up (*q*<0.05)	Spermatogenesis	RNF17, MEI4, MYCBPAP, MOV10L1, SYCP1, CLOCK
	Negative regulation of transcription from RNA polymerase II promoter	EID1, HNF1B, HMGN2, E2F7, E2F8, SOX2, WWC1, MAEL, PAWR, TCF7L2, GLI3, TGFB1, NR1H2, NIPBL, AES, ZKSCAN17, JUND, NR2F6, POU3F3, BHLHE40, ETV6, SIK1, EGR1, ASXL2, EPAS1, FOXJ1, ARID5B, RBL1, CDK6, PLK3, PHF19, HDAC1, HIPK1, BTG2, ID1, JUN, DLX4, SIX1, HIST1H3C, PEG3, NFIB
	Positive regulation of transcription from RNA polymerase II promoter	HNF1B, E2F7, E2F8, JAG1, ZIC1, GLI3, TGFB1, WBP2, HSPH1, TMEM173, NOBOX, IFRD1, TOP2A, AGAP2, EGR1, ARHGEF2, FOXJ1, SOX12, GRHL3, SIX4, PRKD2, DCAF6, NME2, JUN, SIX1, KDM6B, PEG3, SOX2, TCF7L2, ARID2, NR1H2, RGMA, NIPBL, JUND, POU3F3, ETV6, ETV4, ASXL2, KAT2B, EPAS1, ATAD2, IGF2, CAPRIN2, MNAT1, ATF4, HDAC1, BMP7, BMPR1A, NFIB
KO-down (*q*<0.01)	Collagen fibril organization	ADAMTS14, SFRP2, COL3A1, COL1A2, FOXC2, COL1A1, COL5A2, COL5A1
	Embryonic skeletal system morphogenesis	HOXB4, HOXB2, HOXB7, HOXB8, HOXB5, SOX11, HOXB6, HSPG2, FOXC2
	Cell adhesion	TLN2, TNC, PTPRS, COL28A1, CDH1, ITGA3, STAB2, COL16A1, SRC, COL5A1, CASS4, COL7A1, LAMA5, ITGA5, COL6A5, COL6A4, COL6A2, COL6A1, RELN, AATF, EMB, THBS1, THBS2, SPP1
	Anterior/posterior pattern specification	CTNNBIP1, HOXB4, HOXB2, HOXB7, LHX1, HOXB8, SFRP2, HOXB5, HOXB6, HOXB9, TCF15
SC:KO-down *p*<0.05	Spermatogenesis	Prm1, Prm2, Prm3, Klhl10, Odf1, Chd5, Sun5, Ccdc63, Oaz3, Spata20, Galntl5, Atp1a4, Acsbg2
