# Supplementary material for: Gene knockout of Zmym3 in mice arrests spermatogenesis at meiotic metaphase with defects in spindle assembly checkpoint
Source: Cell Death Dis. 2017 Jun 29;8(6):e2910–. doi: 10.1038/cddis.2017.228 (PMC5520888; doi:10.1038/cddis.2017.228)

# Index of Supplemental data

## Supplemental Materials and Methods

## Supplemental Figure Legends

## Supplemental Figures

## Supplementary Tables 1 and 2

## Supplementary Tables 1 (Table S1)

## Differentially expressed genes and GO term enrichments of cultured Zmym3 WT and KO SSCs and isolated spermatocytes by RNA-Sequencing analysis.

## Supplementary Tables 2 (Table S2)

**Primers, sgRNA sequences and antibodies used in this study.**

# Supplemental Materials and Methods

## Generation of KO mice and fertility test

We genotyped four female founders by sequencing the genomic locus targeted by the gRNAs. For all founders, we cloned the PCR-amplified fragments of the locus into the T-vector, transformed E.coli, and then sequenced the bacterial colonies representing various alleles (Supplementary Figure S3a). For founder No. 1, we sequenced 9 colonies and found 3 contained WT *Zmym3* sequence (allele +), 1 contained a KO sequence with a 3-bp deletion (allele 1, a1), and 5 contained a KO sequence with a 106-bp deletion (a2). Because three alleles were identified in one mouse, this founder must be chimeric containing at least two cell types with the following possible genotypes: +/+;a1/a2, or +/a1;+/a2. We crossed this founder female with a WT male and +/a2 heterozygous female offspring were born. Based on these results, we reasoned that the a1/a2 germ cells of the founder were fertile if the founder was +/+;a1/a2 chimera while we knew nothing about the fertility of the homozygous KO female if the founder was of the +/a1;+/a2 genotype. If the founder contained three cell types, one possible genotype was +/+;a1/a1;a2/a2. In this situation, the homologous mutant germ cells were fertile as the +/a2 offspring were derived. For founder No. 2, we sequenced 10 colonies and found that 7 represented a mutant allele with a 128-bp deletion (a3) and 3 represented a mutant allele with a 114-bp deletion (a4). Therefore, the founder was most likely either heterozygous with the a3/a4 genotype or chimeric with the a3/a3;a4/a4 genotype. We crossed this founder female with the WT male and produced both +/a3 and +/a4 female offspring. These results suggested that the KO female was fertile regardless of the genotypes. For the founder No. 3, we sequenced 6 colonies and found that 5 contained a mutant allele with a 159-bp deletion (a5), and 1 contained a mutant allele with a 23-bp deletion (a6). For founder No. 4, we sequenced 9 colonies and found that 4 contained a mutant allele with 2 deletions (one being a 19-bp deletion, and the other being a 14-bp deletion, a7) and 5 contained a mutant allele with a 128-bp deletion (a8). Similarly, these two founders also gave birth to offspring when crossed with WT males. We did not genotype the F1 offspring of No. 3 and No. 4 founders, but reasoned that the founder homozygous females, if they had been generated, were fertile regardless their genotypes. We used the a2/y and a3/y F2 males, which were collectively named -/y (KO) males, for further phenotypic evaluation in the present study. *Zmym3* WT or KO male mice were each housed with two 8-week-old C57BL/6 wild-type female mice for 1 month. Female mice were checked for the presence of a vaginal plug every morning to determine mating activity. The female mice that were vaginal plug positive were separated and observed for 3 weeks for pregnancies. The plugging rate and the number of litter per female were recorded, and fertility rate was calculated.

## Quantification and comparison of spermatogenic cells from WT and KO mice

The quantification of type A spermatogonia (SG-A), intermediate spermatogonia (SG-In), type B spermatogonia (SG-B), leptotene spermatocytes (lepSC), zygotene spermatocytes (zygSC), pachytene spermatocytes (pacSC), diplotene spermatocytes (dipSC); metaphase I spermatocytes (metSC), secondary spermatocytes (secSC), round spermatids (rST), elongating spermatids (eST) was conducted based on histological evaluation. Briefly, on PAS-stained paraffin embedded sections, we identified various spermatogenic cells from the cross sections of seminiferous tubules at certain stages, in which the above spermatogenic cell types were enriched (SG-A from I-II, SG-In from III-IV, SG-B from V-VI, lepSC and rST from VII-VIII, dipSC from XI, zygSC/pacSC from XI-XII, secSC and metSC from XII, and eST from IX-X). For each cell type, at least 9 cross sections from 3 mice were counted. For comparison between WT and KO mice, the numbers per cross section of each cell type were normalized by the number of the WT mice, and the resulting ratios (the one from WT mice being always 1) were compared. For sperm count, cauda epididymis was dissected and immediately minced in 1 ml 1 × PBS solution. Semen was released (15 min at 37℃, 5% CO_2_) from three to five small incisions. Thereafter, the total sperm count was assessed using a hemocytometer.

## Isolation of spermatocytes

Testes of 6-month-old mice were collected, their albuginea were removed. The seminiferous tubules were fragmented and subsequently digested into single cells using a two-step procedure. The first step digestion was carried out using 1 mg/ml type IV collagenase (Sigma) for 5 min at 37 °C to generate shorter fragments of seminiferous tubules. In the second step, the tubule fragments were digested with 0.25% trypsin and 1 mg/ml DNase I (Sigma) at 37 °C for 5 min into single cells. The cells were firstly FACS sorted to collect tetraploid cells which were further sorted into two populations based on their forward scatter (FSC) and side scatter (SSC) features. The population of larger sizes consisted mainly of pachytene spermatocytes of about 80% purity based on the co-immunostaining of SYCP3 and rH2AX were collected.

## TUNEL Assay

Apoptosis assay was performed using the Deadend Fluorometric TUNEL system (Promega) according to the manufacturer’s protocol. Images of TUNEL staining were acquired with a confocal fluorescence microscope (Leica).

## Histology, Immunohistochemistry and Immunofluorescent staining

Fresh mouse testes were fixed in Bouin’s solution (Sigma) or 4% paraformaldehyde overnight at 4℃. Bouin-fixed testis cross-sections were used for H&E staining and periodic acid-Schiff (PAS) staining, and PFA-fixed sections were used for immunohistochemistry and TUNEL staining. H&E staining, PAS staining or immunohistochemistry analyses were carried out following standard protocols. Frozen testis cross-sections and cultured mSSCs were used for immunofluorescent staining. Slides were incubated overnight at 4 ℃ with primary antibodies. On the following day, secondary antibodies were added at room temperature for 2 h. Nuclei were counterstained with DAPI (Sigma). The antibodies used and their dilutions were listed in Table S2.

## Whole-mount immunostaining

Mouse testes were dissected to remove the tunica albuginea, and seminiferous tubules were untangled. Tubules were fixed at paraformaldehyde overnight at 4℃, washed twice with PBS, and blocked with 5% BSA in PBS at 4℃ overnight. The rest of the immunostaining followed the protocol described above.

## Spermatocyte spread

Spermatocyte spreads of testicular samples were performed as previously described by Peters et al.^32^

## Western blot

For Western blot analysis, mSSCs or testis cells were lysed using RIPA lysis buffer (Beyotime Biotechnology). Proteins were separated by 10% SDS-PAGE and electrotransferred onto nitrocellulose membranes. Membranes were then blocked with 5% nonfat milk for 1 h at RT, and then incubated with primary antibodies overnight at 4℃. After washing, membranes were incubated with HRP-conjugated secondary antibodies for 2 h at RT. The proteins were detected using the Supersignal West Pico Chemiluminescent Substrate (Thermo Scientific) before exposure to gel imaging system.

## Figure Legends

## Supplementary Figure S1. Aligned sequences of human and mouse full-length Zmym3. Various domains are color-shaded on the mouse sequence. The encoding exons of the mouse genes are mapped to the protein sequences. This antibody was developed using a synthetic peptide corresponding to amino acids (AAs) 250-300 of the human ZMYM3, which is almost identical to the mouse sequence except for 2 AAs.

## Supplementary Figure S2. ZMYM3 expression profiles in testis and other organs of adult mice. (a) Characterization of the commercial antibody by the co-immunostaining of ZMYM3 and GFP in 293FT cells that were transfected with a plasmid that expresses the GFP-ZMYM3 fusion protein. Scale bar, 50 µm. (b) Alternatively spliced variants of Zmym3 and the lengths and masses of corresponding predicted proteins. Exons encoding the zinc finger (ZNF) domains and the peptide for antibody development are marked. Two groups of protein isoforms were predicted from some validated mRNA sequences, the full-length group (>=1278 AAs, 142 kDa), the shorter group (349~540 AAs, 37~58kDa). (c-d) Zmym3 mRNAs expression profile in six types of mouse spermatogenic cells by RNA-seq (c) and qRT-PCR (n=3) (d) . (e-g) Zmym3 mRNA and protein expression profiles in eight organs of adult mice by RNA-seq (e), qRT-PCR (n=3) (f) and Western blot (g). (h-i) Immunohistochemical analysis of ZMYM3 distribution in ovary of 21-day old mice. Note that ZMYM3 was predominantly present in granulosa cells of all stage follicles and was mainly detected in germ cells of the primordial and primary follicles. The proteins in other stage oocytes were either weakly present or absent.

## Supplementary Figure S3. Morphological comparisons on the testes of the WT and KO mice. (a) Sequencing results of T-A colonies of targeted fragments amplified from the genomes of four female founders. (b) Sizes and histology of testes and epididymides from WT and KO mice at various ages were evaluated visually or by HE staining. Scale bar, 100 µm. (c) TUNEL staining of testis sections from WT and KO mice at ages of 2 months. Scale bar, 50 µm. (d) Histological analyses of sperms from WT and KO mice at ages of 2 months. Scale bar, 100 µm.

## Supplementary Figure S4. (a-g) Staging of the seminiferous tubules of the WT and KO mice by PAS staining. Scale bar, 50 µm.

## Supplementary Figure S5. Co-immunostaining of SYPC3 and γH2AX on the testis sections of the WT and KO mice.

## Supplementay Figure S6. FACS sorting of spermatocytes for RNA-Seq analysis (a) FACS sorting of tetraploid cells from total testicular cells (left) The population of larger size based on their forward scatter (FSC) and side scatter (SSC) features was further sorted from the tetraploid cells (right). (b) The expression of genes involved in SAC according to RNA-seq analysis.

## Supplemental figures


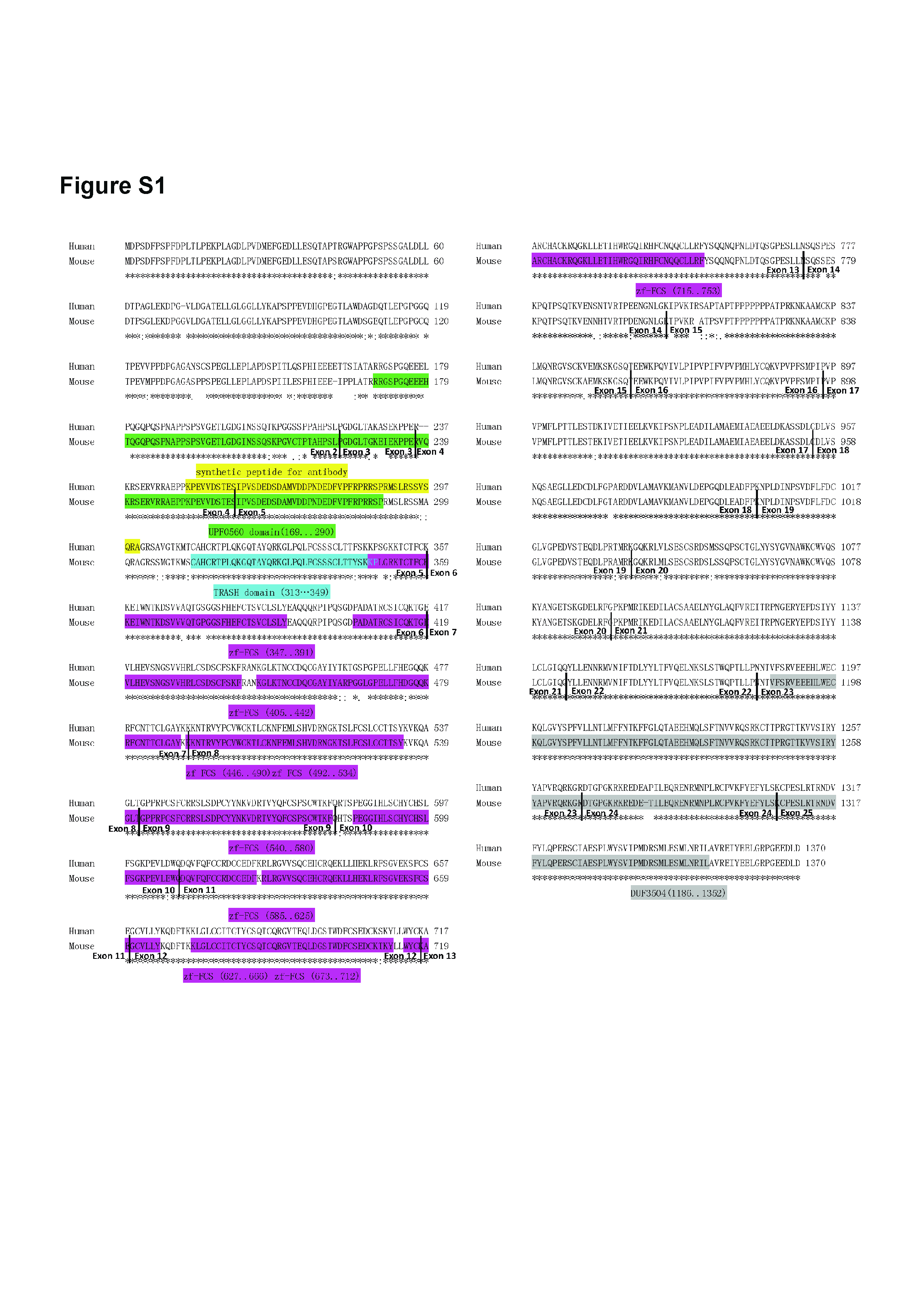


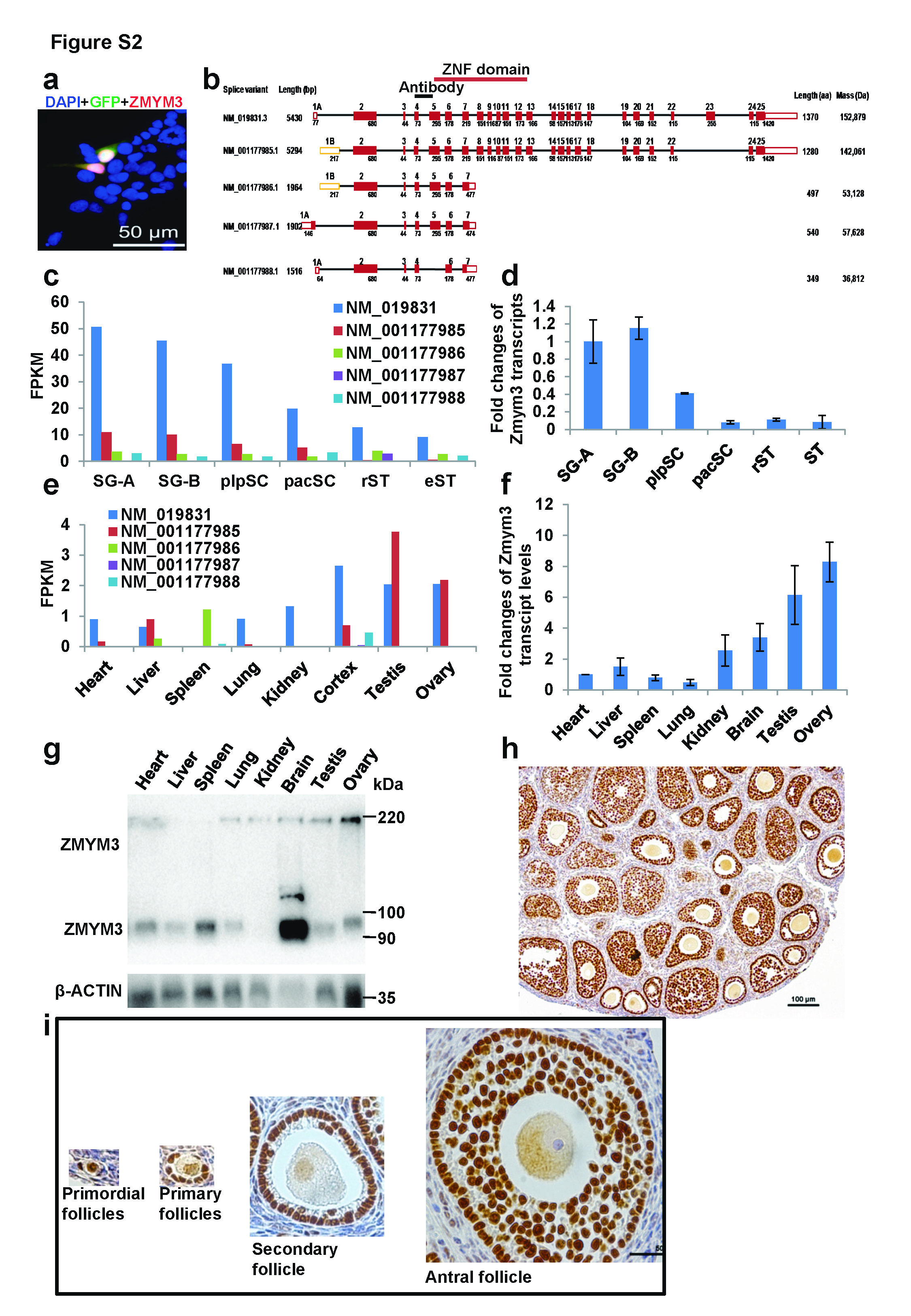


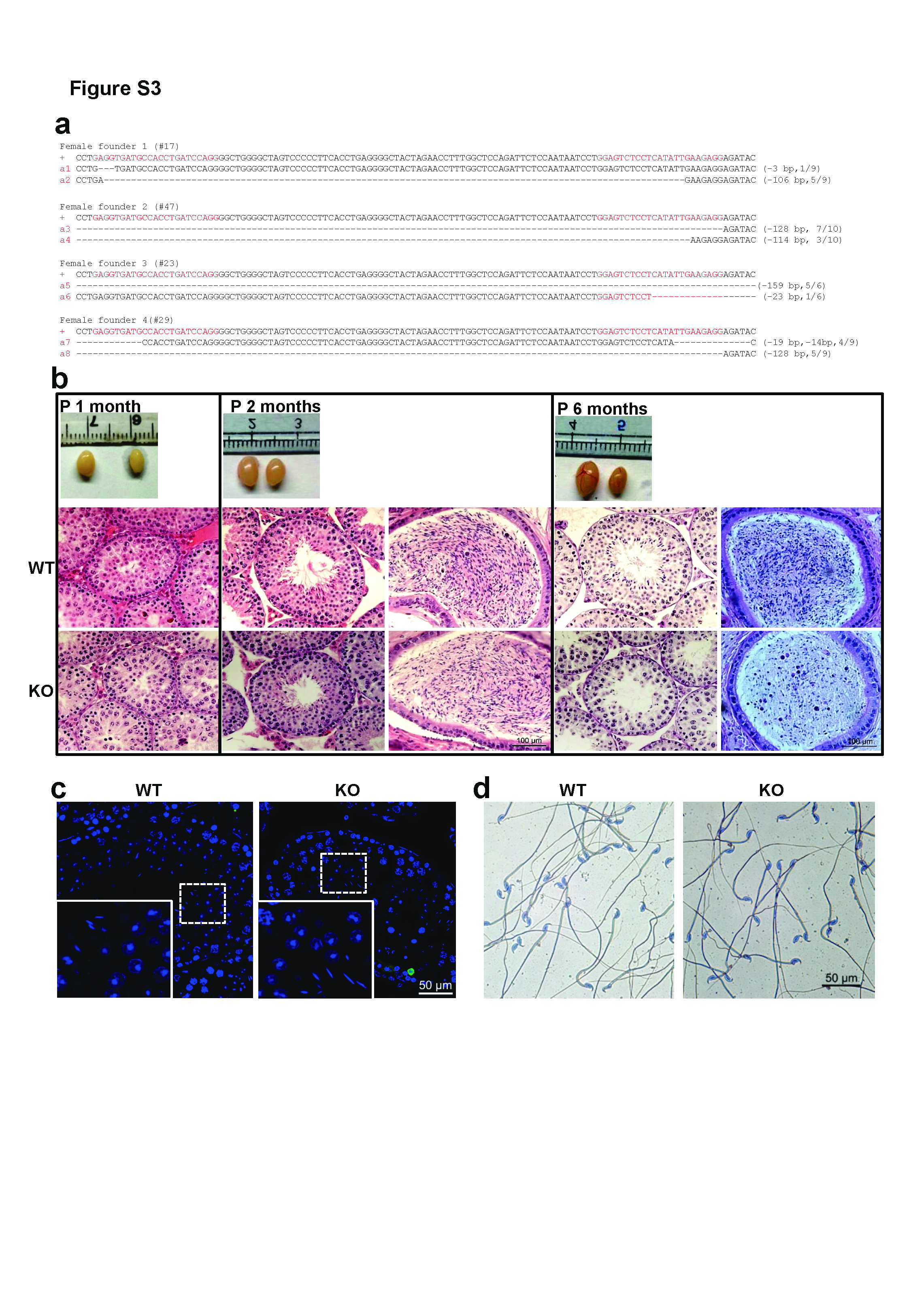


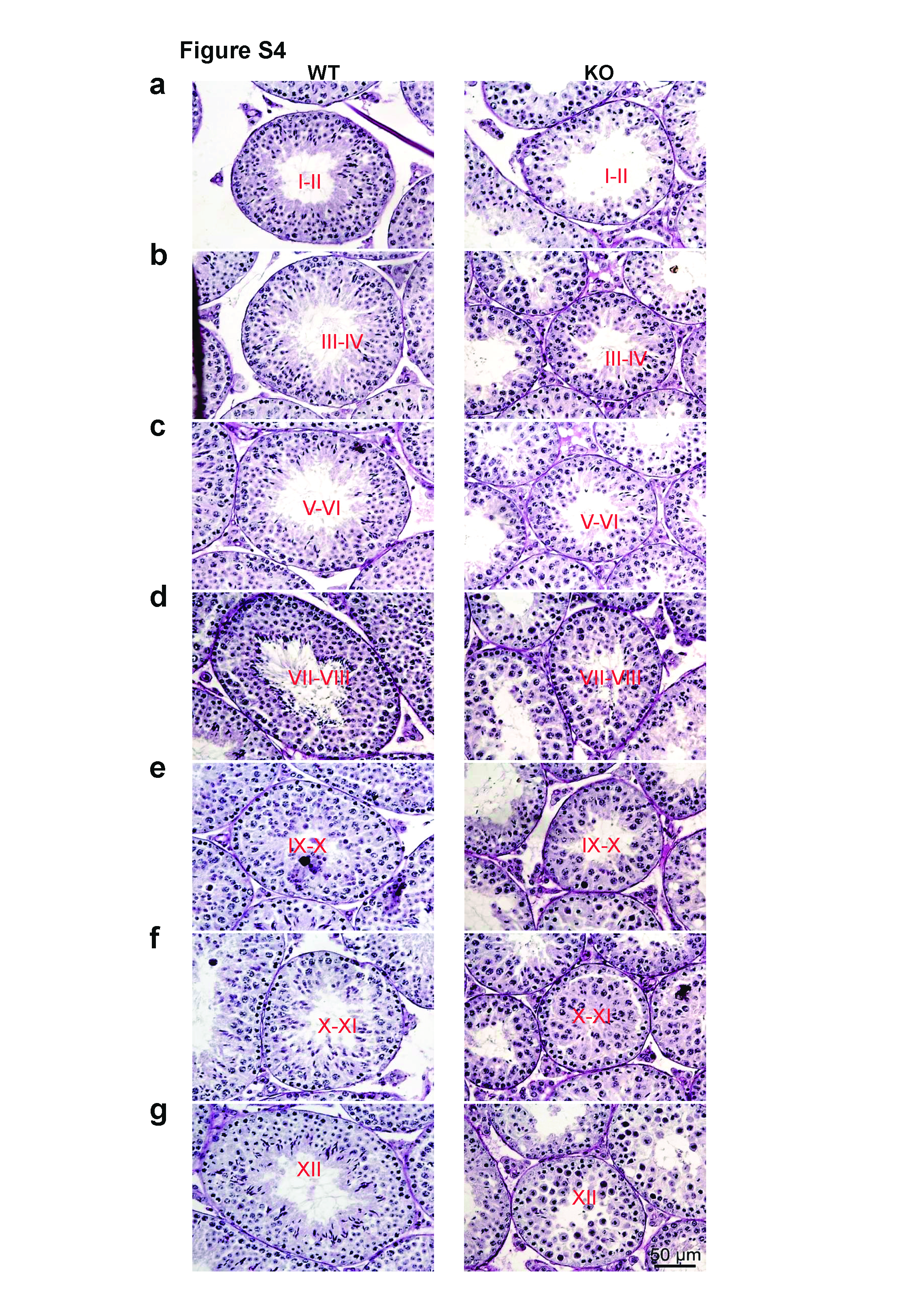


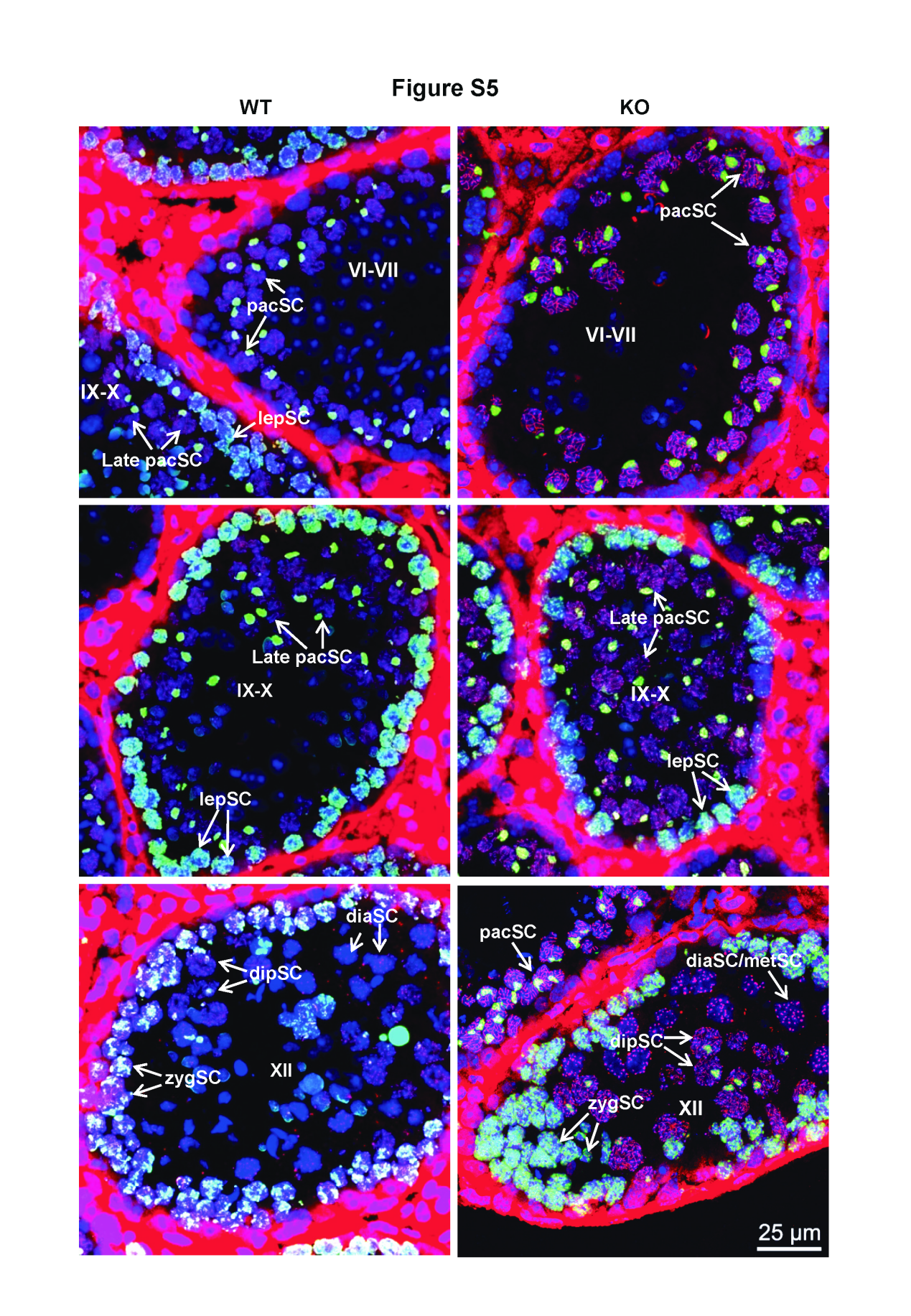


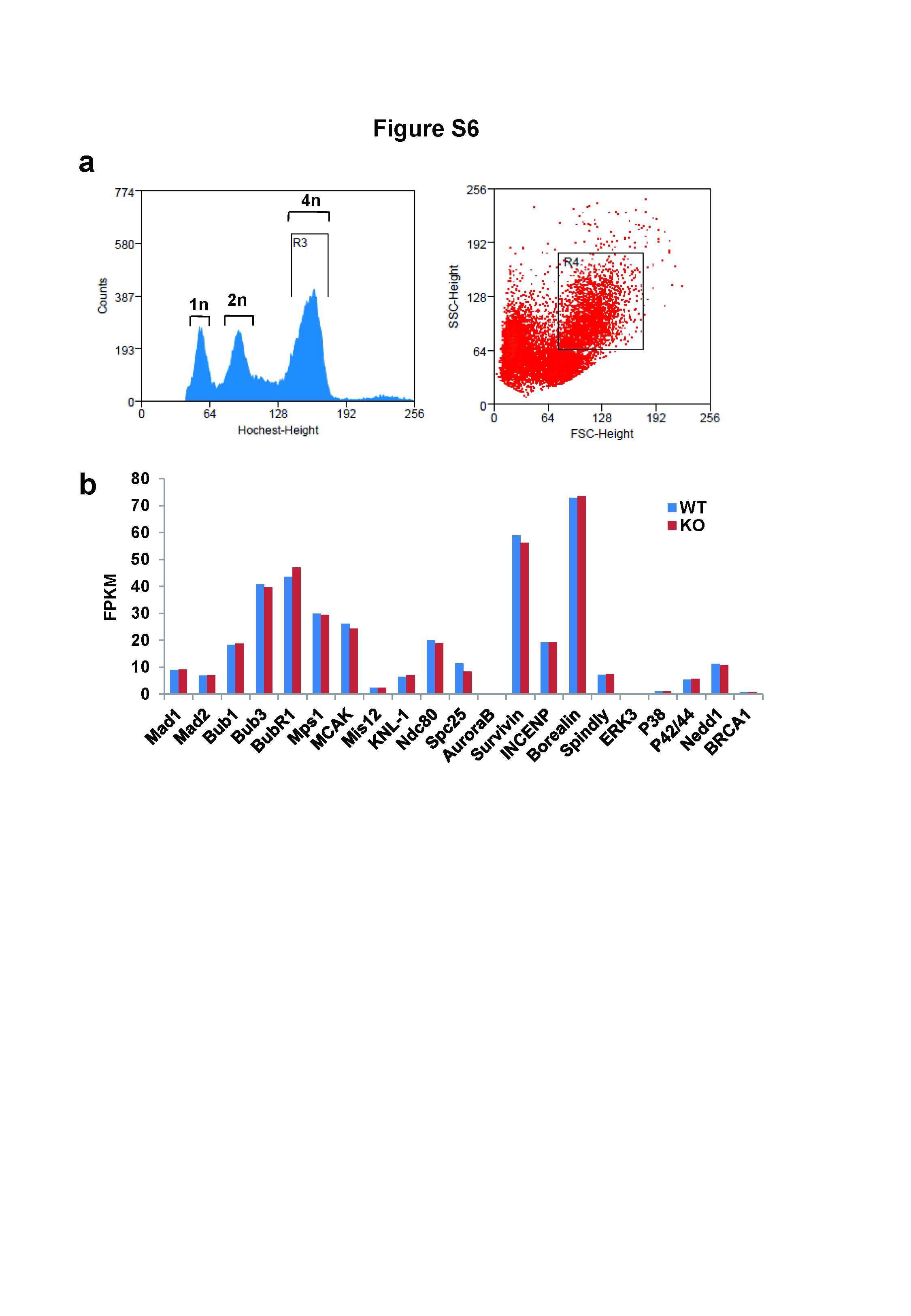

Supplement: Supplementary Information Materials, Methods and Figures [file cddis2017228x1.docx]
